# 

**Published:** 1888-07

**Authors:** 


					﻿Vol VIII.
JULY, 1888.
no.y
IPHE
[JOMCEOP ATHIC pHY£lClA]tf,
A Monthly Journal of Homoeopathic Materia Medica
and Clinical Medicine.
"He is a freeman whom truth makes free, and all are slaves beside.”
"Seek the Truth: come whence it may, cost what it with”
EDITED AND PUBLISHED BY
Edmund j. lee, m. id.,
AND
WALTER M. JAMES, XI. ID.
,	PHILADELPHIA:
No. 1123 SPRUCE STREET.
EOaSTDOlT:
ALFRED HEATH & CO., Sole Agents foe Great Bbitain,
114 Ebury Street, Eaton Square.
4J5f"learZj/ Subscription, $2.50, invariably in advance. Single numbers, 25 cts.
Entered at the Philadelphia Poet-Office as Second-Class Mail Matter.
				

